# Tricuspid isthmus ablation with pulsed-field power by linear catheter: a case report

**DOI:** 10.1093/ehjcr/ytad601

**Published:** 2023-12-08

**Authors:** Xinzhong Li, Long Huang, Jianyong Li, Senlin Huang, Yuegang Wang

**Affiliations:** Department of Cardiology, State Key Laboratory of Organ Failure Research, Nanfang Hospital, Southern Medical University, 1838 North Guangzhou Avenue, Guangzhou 510515, China; Guangdong Provincial Key Laboratory of Shock and Microcirculation; EnChannel Medical Guangzhou Inc, Guangzhou 510005, China; Department of Cardiology, State Key Laboratory of Organ Failure Research, Nanfang Hospital, Southern Medical University, 1838 North Guangzhou Avenue, Guangzhou 510515, China; Guangdong Provincial Key Laboratory of Shock and Microcirculation; Department of Cardiology, State Key Laboratory of Organ Failure Research, Nanfang Hospital, Southern Medical University, 1838 North Guangzhou Avenue, Guangzhou 510515, China; Guangdong Provincial Key Laboratory of Shock and Microcirculation; Department of Cardiology, State Key Laboratory of Organ Failure Research, Nanfang Hospital, Southern Medical University, 1838 North Guangzhou Avenue, Guangzhou 510515, China; Guangdong Provincial Key Laboratory of Shock and Microcirculation

**Keywords:** Tricuspid isthmus, Atrial flutter, Pulsed-field power, Linear catheter, Case report

## Abstract

**Background:**

Pulsed-field ablation using annular or petal-shaped catheters had been proven to be effective for achieving electrical isolation of pulmonary veins in patients with atrial fibrillation. However, the utilization of linear pulse-field power for treating atrial flutter has yet to been documented.

**Case summary:**

In this report, we present a case involving the successful treatment of tricuspid isthmus–dependent atrial flutter treated with a linear pulsed-field catheter. The patient, a 71-year-old male, presents with an electrocardiogram indicating atrial flutter. Subsequent electrophysiological examination reveals typical atrial flutter that is dependent on the cavo-tricuspid isthmus (CTI). This condition is successfully terminated through the application of linear pulsed-field ablation.

**Discussion:**

This case represents a pioneering instance of CTI-dependent atrial flutter ablation utilizing linear pulse-field power. The innovative approach not only effectively treats the patient but also serves as a valuable reference for future applications of linear treatment with pulsed-field ablation.

**Learning points**

- The linear pulsed-field catheter could effectively terminate the tricuspid isthmus–dependent atrial flutter and achieve isthmus block.
- The linear pulsed-field power could be expected to become the promising means of linear ablation.

## Introduction

Pulsed-field ablation has been widely utilized with annular or petal-shaped catheters for the purpose of electrically isolating pulmonary veins in atrial fibrillation.^1,2^ However, the use of linear pulse-field power in the treatment of atrial flutter has not been previously reported. In this article, we present a case involving the treatment of tricuspid isthmus–dependent atrial flutter using a linear pulsed-field catheter.

## Summary figure

**Figure:**
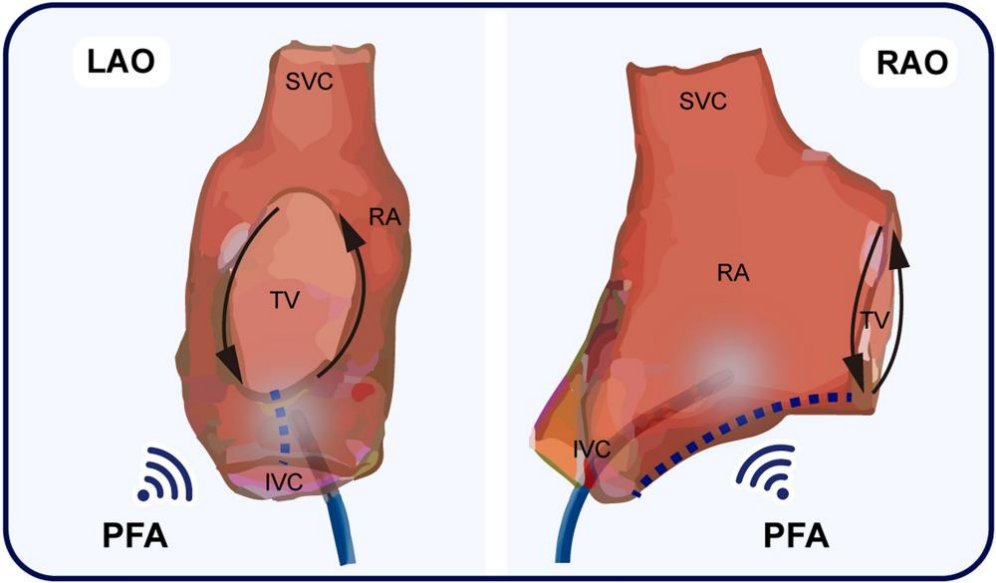


## Case report

**Figure 1:**
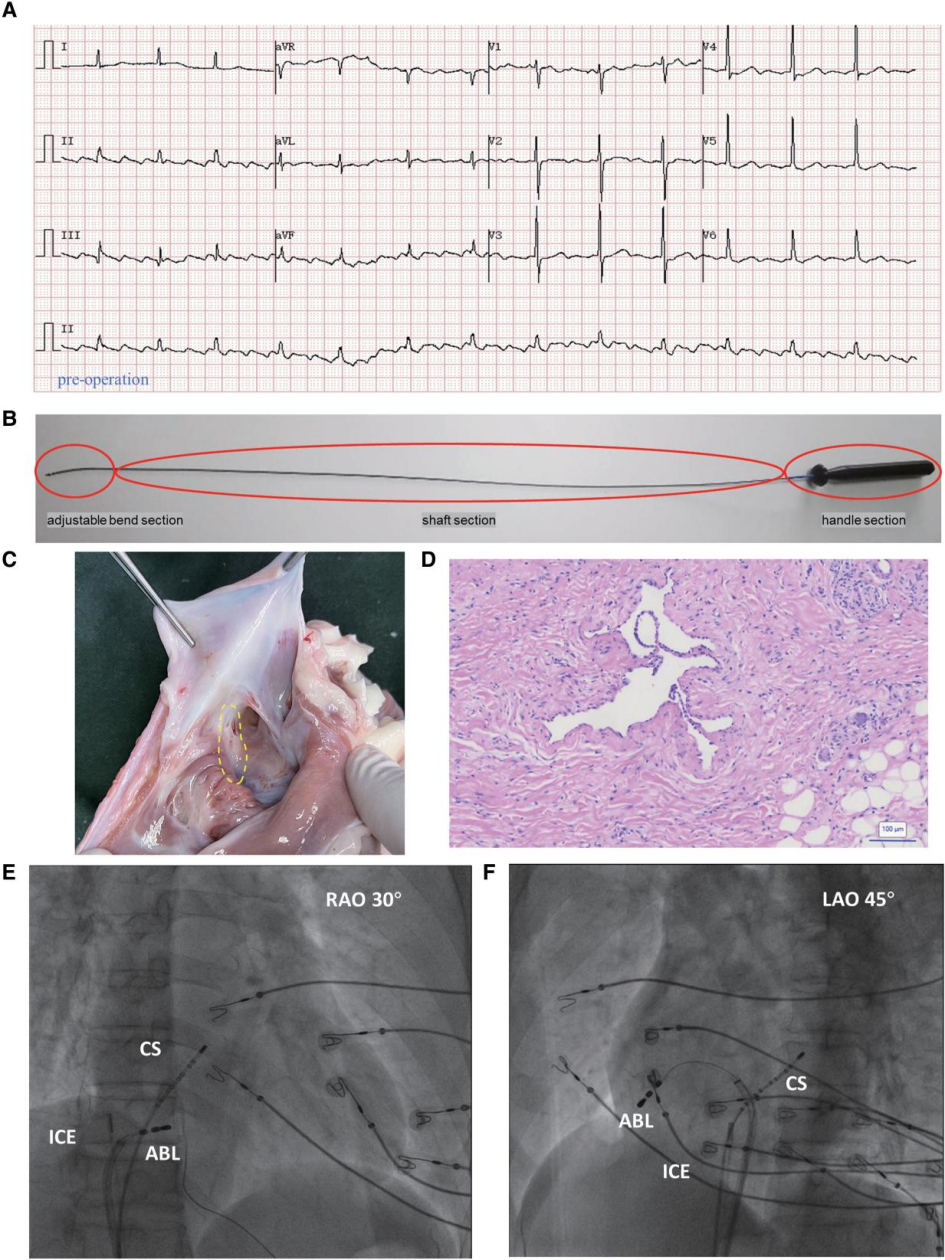
(*A*) The electrocardiogram of atrial flutter before operation. (*B*) The image of linear pulse-field ablation catheter with red circles marking different parts of the catheter. (*C*) Damage caused by the linear catheter ablation on the cavo-tricuspid isthmus of pigs. The yellow dotted line represented the damaged area. (*D*) Haematoxylin and eosin staining of the damaged area showed no myocardial fibres (bar = 100 µm). (*E* and *F*) The X-ray images when arrhythmia was terminated and bidirectional block was achieved. CS, coronary sinus; ICE, intracavitary ultrasound; ABL, ablation catheter.

**Figure 2:**
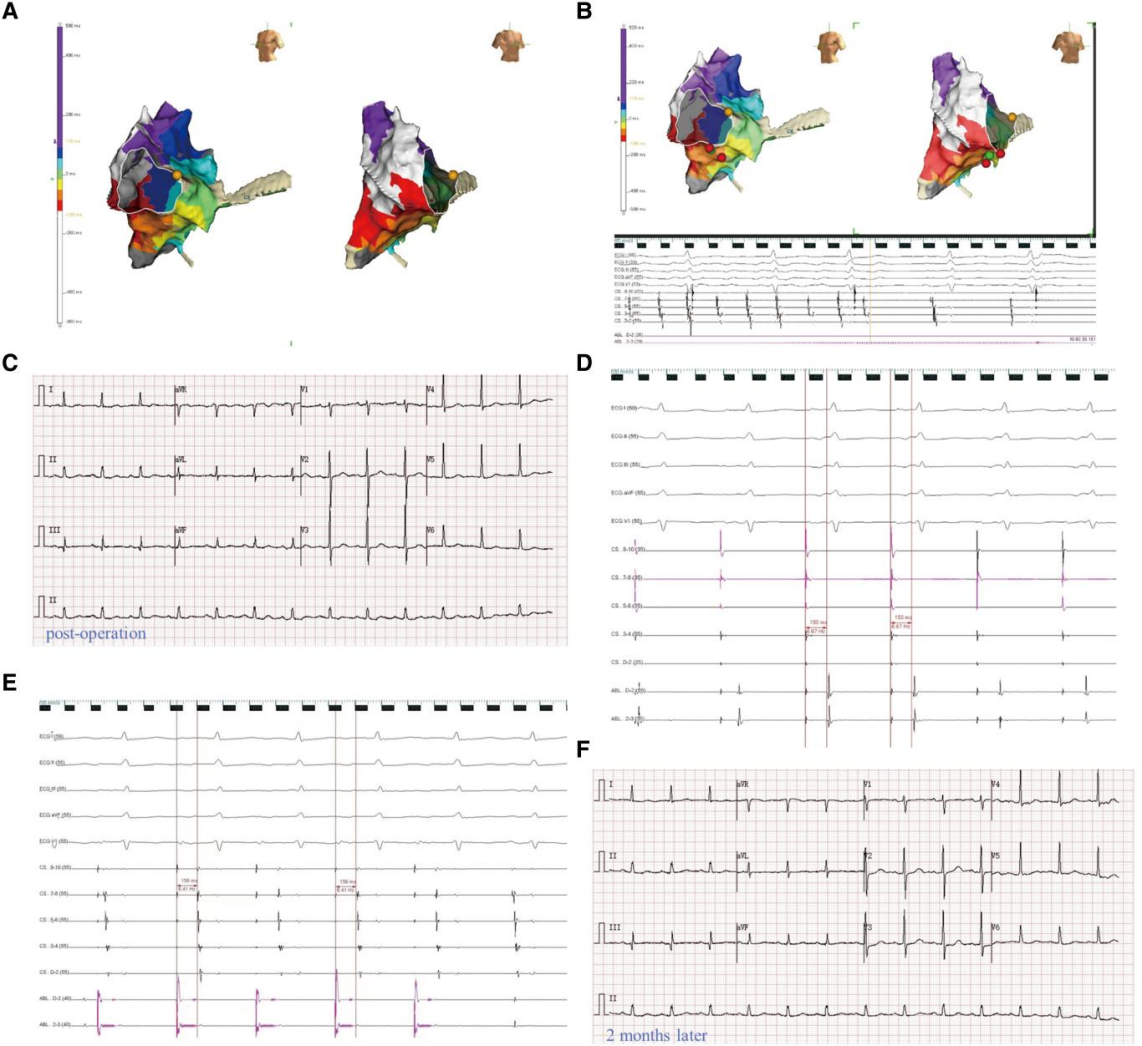
(*A*) Red, orange, yellow, green, cyan, blue, and violet represented the sequence of tachycardia in the isochronous diagram of the left and right anterior oblique, which indicated the electroanatomical mapping with tricuspid annular reverse clock return. (*B* and *C*) The ablation of linear pulse-field ablation terminated the tachycardia. (*D* and *E*) The catheter was placed on the other side of the block line, and the pacing time from CS90 to ABL12 was 150 ms, while the pacing time from ABL12 to CS90 was 156 ms. (*F*) The electrocardiogram 2 months after ablation indicated sinus rhythm.

The electrocardiogram (ECG) reveals atrial flutter (*Figure 1A*) in a 71-year-old patient with a history of coronary artery bypass surgery. At the outset of the procedure, we administer intravenous midazolam (0.03 mg/kg) and propofol (0.05 mg/kg) slowly, followed by a continuous infusion via a syringe pump at a rate of 4 mg/kg/h. Dosage adjustments are made as necessary in response to the patient’s movements or reported discomfort. The scheduled procedure involves pulsed-field ablation using a linear ablation catheter (PulseLine, PL03F07N, EnChannel Medical Guangzhou Inc) and a pulsed electric field instrument (NanoAblate, PG-01, EnChannel Medical Guangzhou Inc), which has been shown to cause sustained damage in animal studies (*Figure 1B–F*). The catheter used has a diameter of 2.33 mm, an effective length of 1100 mm, and a conducting area of 4618 mm^2^. The electroanatomical mapping of the tachycardia as well as entrainment manoeuvres indicates typical cavo-tricuspid isthmus (CTI)–dependent atrial flutter (*Figure 2A*). After beginning the continuous infusion with nitroglycerine (5 μg/min), one linear pulsed-field ablation at the tricuspid isthmus terminates the atrial flutter (*Figure 2B* and *C*). Subsequently, atrial activation during septal and lateral atrial pacing confirms the bidirectional CTI block (*Figure 2D* and *E*). Over the following 2 months, the patient remains free of arrhythmias (*Figure 2F*).

## Discussion

It has been known that CTI ablation is an effective way to terminate typical atrial flutter. In this case, we successfully achieve bidirectional isthmus block and terminate the tachycardia using a linear pulsed-field catheter.

In previous studies of pulsed-field ablation in paroxysmal atrial fibrillation, petal-shaped catheters are tried to isolate the apical, mitral, and tricuspid isthmic lines with promising results.^3,4^ Moreover, in a case of mitral isthmus–dependent atrial flutter, bidirectional isthmus block is achieved through pulsed-field ablation using a petal-shaped catheter.^5^ These findings suggest that pulsed-field ablation can also accomplish linear isolation. However, the non-linear catheter poses challenges in achieving effective tissue attachment, hindering its clinical application. In contrast, our use of a linear catheter, which has previously been demonstrated to be safe in domestic pigs, marks its inaugural application in atrial flutter and successful completion of isthmus bidirectional block. This experience provides valuable insights for future application of linear pulsed-field power. Furthermore, a previous study has identified the potential for coronary spasm during pulsed-field ablation for the mitral isthmus,^6^ leading us to proactively administer nitroglycerine and pay attention to ST-segment changes in ECG to prevent this adverse reaction.

## Conclusion

The linear pulsed-field power can be used to achieve effective tricuspid isthmus block, which suggests that pulsed-field energy can be applied to a wider range of linear ablation.

## Data Availability

The data underlying this article will be shared upon reasonable request to the corresponding author.

## Lead author biography

Deputy chief physician, doctor, visiting scholar of OHSU Department of Cardiovascular Disease, arrhythmia training instructor of the National Health Commission, and director of Nanfang Hospital Training Base. Member of the Electrophysiology Group, Pacing and Electrophysiology Branch, Chinese Medical Association; Standing Committee of Cardiac Pacing and Electrophysiology Branch of Guangdong Medical Association and leader of Sudden Death Prevention Group; member of the Committee of Pacing and Electrophysiology Branch of Guangdong Medical Association; and member of the Cardiovascular Disease Branch of Guangdong Medical Association. Have now finished >3000 cases of PVC, VT, and atrial arrhythmia with EnSite Velocity or CARTO3 system.

**Consent:** The authors confirm that written consent for submission and publication of this case report including image(s) and associated text has been obtained from the patient in line with COPE guidance.

**Funding:** This work is supported by grants awarded to X.L. from the National Natural Science Foundation of China (no. 82000248) and Guangzhou Municipal Science and Technology Project (no. 2023A04J2272) as well as grants awarded to Y.W. from the Natural Science Foundation of Guangdong Province (no. 2023A1515011833).
